# Acquired deficiency of prothrombin complex coagulation factors in major bleeding is a therapeutic indication of 4‐factor prothrombin complex concentrates

**DOI:** 10.1111/tme.12723

**Published:** 2020-09-28

**Authors:** Christian J. Wiedermann

**Affiliations:** ^1^ Institute of Public Health, Medical Decision Making and HTA University of Health Sciences, Medical Informatics and Technology Hall (Tyrol) Austria

Dear Sir,

Recently, Marcos‐Jubilar et al^1^ reported that 4‐factor prothrombin complex concentrate (4F‐PCC) was effective as adjuvant treatment with an acceptable safety profile in the approved emergent reversal of vitamin K antagonist indication, as well as in the refractory coagulopathy associated with major bleeding. In the publication of their observational study performed in two Spanish tertiary university hospitals, coagulopathy associated with major bleeding was categorised as an off‐label indication of 4F‐PCCs.^1^

Typically, 4F‐PCC preparations are licensed for the treatment of bleeding in acquired deficiency of the prothrombin complex coagulation factors. In the package inserts in Europe, including Spain and the United Kingdom, deficiencies caused by treatment with vitamin K antagonists, or in case of overdose of vitamin K antagonists, when rapid correction of the deficiency is required, are listed as example indications for 4F‐PCCs but without excluding other types of acquired deficiency of the prothrombin complex coagulation factors, such as in major perioperative bleeding. Major bleeding can involve acquired coagulation abnormalities with deficiency of prothrombin complex coagulation factors secondary to haemorrhage, haemodilution or haemostatic factor consumption.^2^ Thus, in the publication by Marcos‐Jubilar et al,^1^ refractory coagulopathy associated with major bleeding should not be classified as an off‐label indication of 4F‐PCC.

## CONFLICT OF INTEREST

C. J. W. received lecture fees from and served as a consultant to CSL Behring (Germany) and Grifols (Spain).

## AUTHOR CONTRIBUTION

C. J. W. is the sole author. No other person was involved in the conception and design, the analysis and interpretation of the data, the drafting of the paper, or revising it critically for intellectual content.

## References

[1] Marcos‐Jubilar M , García Erce JA , Martínez‐Calle N , Páramo JA , Martínez Virto A , Quintana‐Díaz M . Safety and effectiveness of a prothrombin complex concentrate in approved and off‐label indications. Transfus Med. 2019;29:268‐274. 31347218 10.1111/tme.12621

[2] Ghadimi K , Levy JH , Welsby IJ . Perioperative management of the bleeding patient. Br J Anaesth. 2016;117(suppl 3):iii18‐iii30. 27940453 10.1093/bja/aew358 PMC5155545

